# Host Species Determines the Composition of the Prokaryotic Microbiota in *Phlebotomus* Sandflies

**DOI:** 10.3390/pathogens9060428

**Published:** 2020-05-29

**Authors:** Christos Papadopoulos, Panagiotis A. Karas, Sotirios Vasileiadis, Panagiota Ligda, Anastasios Saratsis, Smaragda Sotiraki, Dimitrios G. Karpouzas

**Affiliations:** 1 Laboratory of Plant and Environmental Biotechnology, Department of Biochemistry and Biotechnology, University of Thessaly, 41500 Larissa, Greece; papadopoulos.chr@hotmail.com (C.P.); karaspan@yahoo.com (P.A.K.); vasiliad@gmail.com (S.V.); 2 Group of Parasitology, Veterinary Research Institute, Hellenic Agricultural Organization-Demeter, 57100 Thessaloniki, Greece; giota.lig@hotmail.com (P.L.); saratsis@vri.gr (A.S.)

**Keywords:** sandflies, *Phlebotomus*, bacteria, archaea, microbiota, host genotype

## Abstract

Phlebotomine sandflies are vectors of the humans’ and mammals’ parasite *Leishmania* spp. Although the role of gut microbiome in the biological cycle of insects is acknowledged, we still know little about the factors modulating the composition of the gut microbiota of sandflies. We tested whether host species impose a strong structural effect on the gut microbiota of *Phlebotomus* spp. Sandflies were collected from the island of Leros, Greece, and classified to *P. papatasi, P. neglectus, P. tobbi,* and *P. similis*, all being negative to *Leishmania* spp. The prokaryotic gut microbiota was determined via 16S rRNA gene amplicon sequencing. *Phlebotomus* species supported distinct microbial communities (*p* < 0.001). *P. papatasi* microbiota was the most distinct over-dominated by three *Spiroplasma, Wolbachia* and *Paenibacillus* operational taxonomic units (OTUs), while another *Wolbachia* OTU prevailed in *P. neglectus*. Conversely, the microbiota of *P. tobbi* and *P. similis* was composed of several less dominant OTUs. Archaea showed low presence with the dominant OTUs belonging to methanogenic Euryarcheota, ammonia-oxidizing Thaumarcheota, and Nanoarchaeota. We provide first insights into the composition of the bacterial and archaeal community of *Phlebotomus* sandflies and showed that, in the absence of *Leishmania,* host genotype is the major modulator of *Phlebotomus* sandfly gut microbiota.

## 1. Introduction

Phlebotomine sandflies (Diptera: Psychodidae, Phlebotominae) are insects of global health importance. This is based on their versatility as vectors of human pathogens including viruses (*Phleboviruses*) [1,2], bacteria (*Bartonella bacilliformis*) [3] and protozoa like *Leishmania* spp. [4]. In Europe and mainly in the Mediterranean region, sandflies of the genus *Phlebotomus* have been incriminated as vectors of *Leishmania* [5] transmitted by the bite of an infected female sandfly [6].

Collective field studies in the Mediterranean region [7] and neighboring countries like Albania [8] and Serbia [9,10], verified the presence of several *Phlebotomus* species like *P. neglectus, P. tobbi, P. balkanicus, P. ariasi, P. perfiliewi, P. perniciosus, P. kandelakii, P. mascitii, P. alexandri, P. sergenti. P. similis, P. simici, P. patasi,* all being proven or potential vectors of *Leishmania*. In Greece, monitoring surveys identified *P. neglectus* (32.8% of the population captured in the study), *P. similis* (30.3%), *P. tobbi* (16.7%), and *P. perfiliewi* (15.9%) in Ionian islands, whereas *P. simici* (50%), *P. neglectus* (24.5%), and *P. tobbi* (9.6%) predominated in the North Greece mainland (Xanthopoulou et al.) [11]. Recent studies in 11 islands in the Aegean Sea detected a rich sandfly fauna comprised of *P. neglectus, P. tobbi, P. similis, P. simici, P. perfiliewi, P. alexandri* and *P. papatasi* [12]. 

Insects live in association and interact with a diverse microbiota. These interactions have serious implications for the fitness, ecology and evolution of insects. Gut microbiota provides beneficial services to their insect host. They facilitate the nutrition of insects which feed on nutrient-deficient diets enabling their survival in oligotrophic environments [13]. Endosymbiotic vertically inherited bacteria like *Wolbachia* and *Spiroplasma* manipulate the reproduction system of their hosts to prevail in their population over endosymbiont-free individuals [14], alter the sex-ratio of the population via production of male-killing toxins [15] or protect their hosts (i.e., *Drosophila melanogaster, Aeges aegypti*) from parasitic wasps [16] and other insect parasites [17]. Insects also utilize microbiome as a rapid evolution mechanism to adapt to rapidly changing environmental conditions [18]. In this context, a symbiotic *Citrobacter* carrying an organophosphate hydrolase conferred resistance in *Bactocera dorsalis* to organophosphate insecticides [19]. Conversely, insect symbiotic bacteria could be detrimental to their hosts by conferring pathogenesis (i.e., *Spiroplasma* and honeybees) [20] or by interacting with plant defense systems exacerbating their effect on insect pests [21]. 

Although we have just started to appreciate the role of microorganisms, the association between the composition of insect gut microbiome and phylogenetic or ecologic traits of the host is not well defined yet. Several studies have investigated the structural role of diet, the local ecosystem and climatic conditions, the phylogeny and the life stage on insect gut microbiota. Kolasa et al. [22] showed that the composition of the gut microbial community in beetles was better explained by trophic guild. Muturi et al. [23] showed that sampling location exhibited the strongest structural effects on the gut microbial community of mosquitos, while Sanders et al. [24] showed a strong influence of host species in the composition of the bacterial community of turtle ants. In a survey including insects from various trophic guilds and taxa, Yun et al. [25] suggested that host habitat, diet, developmental stage and phylogeny, all contributed to structuring insect gut microbiota. 

Special attention has been given to the microbiota of insects which are vectors of important parasites. Sandfly gut microbiota can be modulated (i) by their feeding habits: larvae feeding on soil dead organic matter, while female adults get blood and sugar meals (plant-fed) and (ii) through interactions with transmitting parasites [26,27]. To date the gut microbiota of *Phlebotomus* sandflies has been studied using culture-dependent approaches [28,29], while recent studies on *Lutzomyia longipalpis* have used culture-independent approaches [30,31]. The gut bacterial community of sandflies is dominated by Proteobacteria and Firmicutes. Initial studies showed that certain members of the sandfly gut microbiota exerted a negative effect on the development of *Leishmania* [32,33]. This was challenged recently by Louradour et al. [34] who showed that the gut microbiota enhances the survival of *Leishmania* by modulating optimal osmotic conditions for promastigotes in the midgut. Further studies by Dey et al. [35] suggested that gut microbes of *L. longipalpis* are egested into the host skin alongside *Leishmania* triggering neutrophil infiltration and facilitating parasite establishment. To date most studies have looked into the gut microbiota of sandflies through the prism of its interaction with the transmitting parasite or as a pool of symbiotic organisms with the potential to be used in paratransgenesis control strategies. However, we still lack data about the role of phylogeny on structuring the gut microbiota of sandflies. This is especially true for sandflies of the genus *Phlebotomus*, whose associated microbiota has been studied using less informative culture-dependent methods. 

We posit that host phylogeny is a main determinant of the composition of the gut microbiota of *Phlebotomus* sandflies. To test this hypothesis, we collected wild sandfly specimens (over a two-year period) from a rather isolated population in a sandfly endemic area. Morphologically identified *Phlebotomus* species were used to perform amplicon sequencing analysis of the 16S rRNA gene of bacteria and archaea, particularly for the later whose presence in insect symbiome is underscored [36]. Specimens analyzed were all *Leishmania*-free, as verified by quantitative polymerase chain reaction (q-PCR), allowing us to study the structural role of sandfly phylogeny on the gut microbiota in the absence of any other interacting factors (i.e., *Leishmania* presence).

## 2. Results

### 2.1. Morphological Identification of Phlebotomus Species 

In total 1235 sandflies belonging to 10 different species were collected from the island of Leros, Greece during the two-year sampling period (2017–2018), 566 (45.8%) males and 669 (54.2%) females. Collected sandflies were assigned to the following species in descending prevalence: *P. papatasi* (51.5%), *P. neglectus* (19.8%), *P. tobbi* (17.1%), *Sergentomyia minuta* (3.3%) *P. similis* (2.7%), *P. simici* (2.2%), *P. perfiliewi* (1.8%), *S. dentata* (1.4%), *P. mascitti* (0.2%) and *P. alexandri* (0.1%). Upon homogenization 37, 15, 10 and 10 samples of *P. papatasi*, *P. neglectus, P. tobbi* and *P. similis*, constituting the most prevalent *Phlebotomus* species, were processed for DNA extraction. From the sandflies collected none was found positive to *L. infantum* according to our q-PCR test (Supplementary Materials Figure S1). All these samples we further processed for determination of their prokaryotic gut microbiome.

### 2.2. The Composition of the Bacterial and Archaeal Microbiome in Sand Flies

From the 16S rRNA amplicon sequencing of all samples we obtained 1,503,942 quality sequences (19,281 per sample, range 2200–82,059) which were assigned to 3762 and 22 operational taxonomic units (OTUs) for bacteria and archaea, respectively. Our sequencing effort provided adequate coverage of the microbial diversity on the gut of sandflies as suggested by the rarefaction curves, which reached a plateau in all samples tested (Supplementary Materials Figure S2).

**Figure 1:**
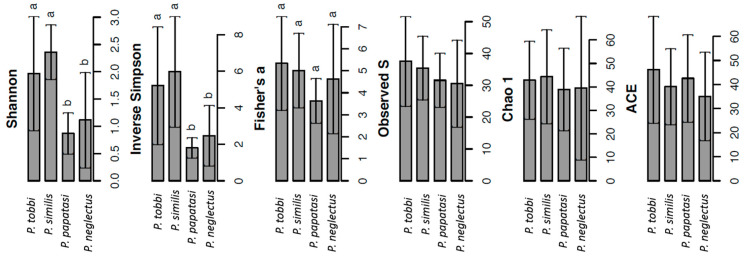
Analysis of the α-diversity of bacteria in the gut of *P. tobbi, P. similis, P. papatasi* and *P. neglectus* based on the calculation of the indices Shannon, Inverse Simpson, Fisher’s alpha, Evenness (S), Chao 1 and Abundance-based Coverage Estimator (ACE). Error bars represent the standard error. Within each index, bars designated by the same letter are not significantly different at the 5% level.

We observed significant differences in the α-diversity of bacteria among species. Hence, significantly lower values (*p* < 0.05) of Shannon and inverse Simpson diversity indices were observed in *P. papatasi* and *P. neglectus* compared to the other two species (Figure 1). In contrast, we did not observe significant differences between the different *Phlebotomus* species in the other α-diversity indices obtained. 

**Figure 2:**
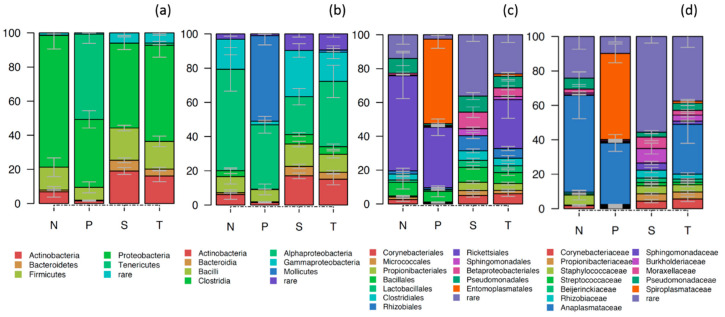
Stacked bar plot showing the relative abundance of bacteria in the gut of the different *Phlebotomus* species (N: *P. neglectus*, P: *P. papatasi*, S: *P. similis* and T: *P. tobbi*) presented at the phylum (**a**), class (**b**), order (**c**) and genus (**d**) taxonomic level. Error bars represent the standard deviation of three biological replicates. Taxa that participated with less than 1% in 80% or more of the samples analysed were grouped as “Rare”.

The gut bacterial community of *Phlebotomus* was dominated by Proteobacteria (55.8%, 39.8%–77.3%), mostly of the classes of α-proteobacteria (39.3%) and γ-proteobacteria (15.9%), Firmicutes (14%, 7.5%–18.9%), Tenericutes (12.9%, 0.01%–50.0%), Actinobacteria (10.9%, 1.5%–19.0%) and Bacteroidetes (3.0%, 0.5%–6.5%) (Figure 2). The large representation of α-proteobacteria and Tenericutes was associated, almost entirely, with the presence of endosymbiotic *Wolbachia* and *Spiroplasma,* respectively. Non endosymbiotic proteobacteria like *Pseudomonas* (3.2%), *Acinetobacter* (2.3%), *Methylobacterium* (1.1%), Firmicutes like *Staphylococcus* (3.5%) and Actinobacteria like *Cutibacterium* (2.2%) were also present at lower relative abundance (RA) (<5%). The core microbiota of *Phlebotomus* species (all OTUs that participated with at least 0.1% RA in at least the 50% of the samples analysed) was composed of 14 OTUs belonging to α-proteobacteria (*Wolbachia, Sphingomonadaceae, Rhizobiaceae*), γ-protebacteria (*Pseudomonas, Acinetobacter*), Firmicutes (*Staphylococcus*), Actinobacteria (*Corynebacteriaceae, Cutibacterium, Streptococcus*) and Tenericutes (*Spiroplasma*) (Supplementary Materials Figure S3). 

**Figure 3:**
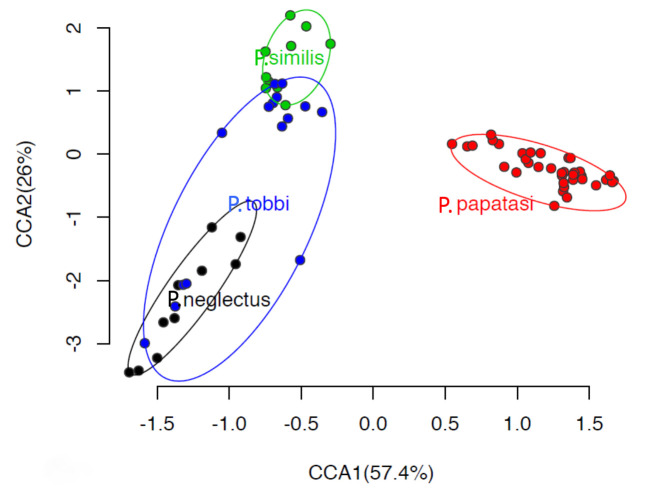
Canonical correspondence analysis (CCA) of the bacterial community in the gut of *P. papatasi, P. tobbi, P. similis* and *P. neglectus.* Ellipses encompass all samples of the same *Phlebotomus* species. The model testing the host genotype effect on the bacterial community structure was significant (*p* < 0.001) and explained 45.1% of the total variance.

At the β-diversity level, canonical correspondence analysis (CCA) showed that *P. papatasi* supported a largely different bacterial community which was clearly separated along CCA1 from the bacterial communities (*p* < 0.001) in the other three *Phlebotomus* species which formed a second group (Figure 3). Within this second group, samples from *P. similis* and *P. neglectus* were also significantly separated (*p* < 0.01) along CCA2 (explaining 26% of the variance), while a weaker but still significant separation (*p* < 0.05) was evident between *P. tobbi* and the other two genera.

**Figure 4:**
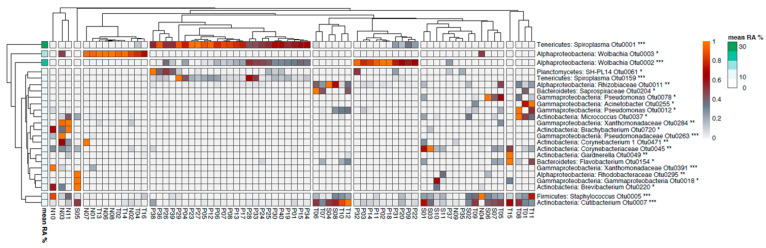
Heatmap analysis showing operational taxonomic units (OTUs) whose relative abundance exhibits significant correlation with certain *Phlebotomus* species. The level of significance is denoted by asterisks as follows: * *p* < 0.05, ** *p* < 0.01, *** *p* < 0.001). N: *P. neglectus*; P: *P. papatasi*; T: *P. tobbi*; S: *P. similis.*

**Figure 5:**
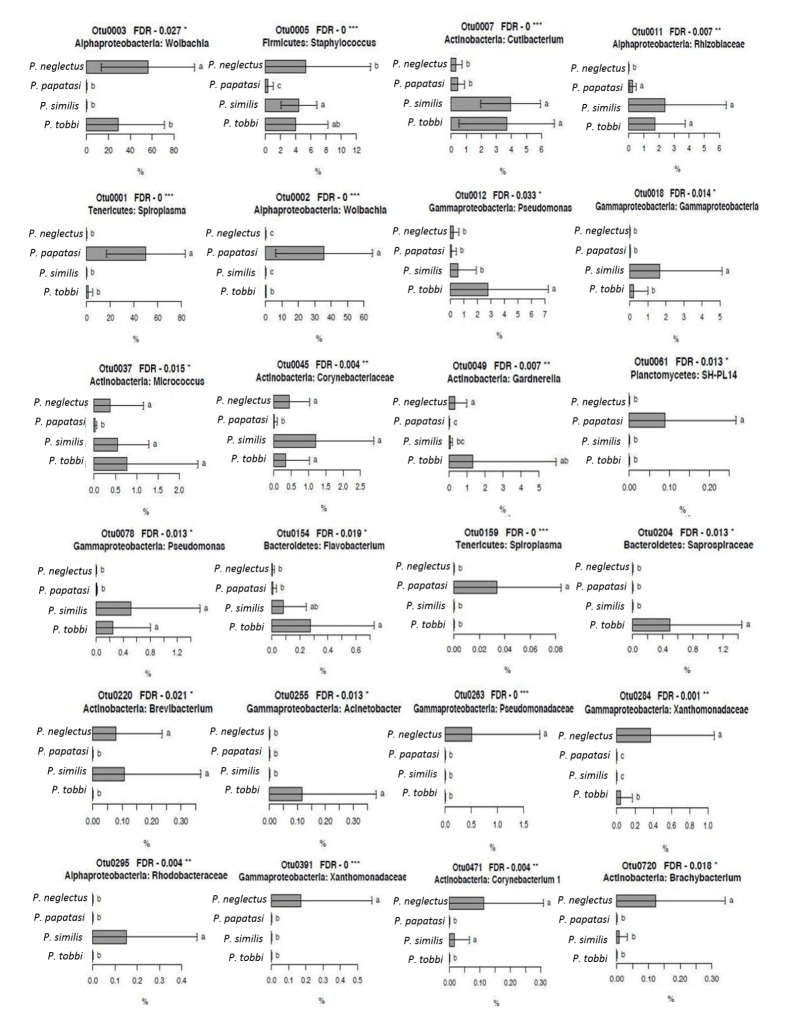
The relative abundance (RA) of bacterial OTUs showing species-specific patterns on the different *Phlebotomus* species. Error bars represent standard errors. For each OTU, bars designated by the same letter are not significantly different. * *p* < 0.05, ** *p* < 0.01, *** *p* < 0.001.

**Figure 6:**
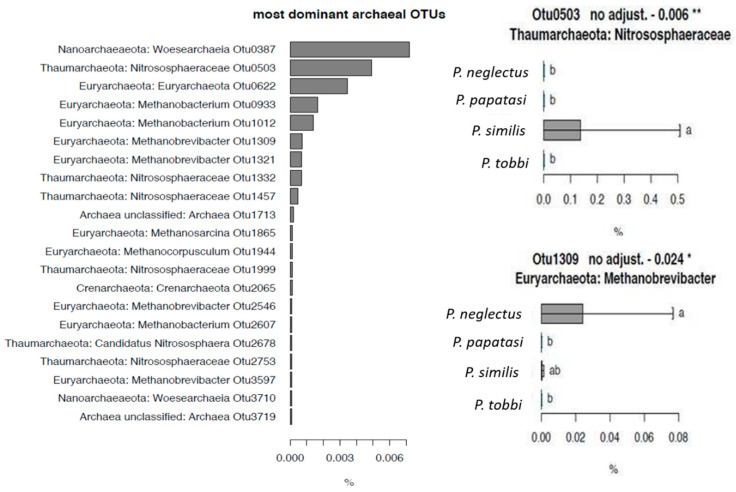
(**a**) The relative abundance (RA) of the most dominant archaeal OTUs detected in the sandfly samples analysed and (**b**) The relative abundance of archaeal OTUs 503 and 1309, which showed species-specific patterns, on the different *Phlebotomus* species studied. Error bars represent standard errors while bars designated by the same letter are not significantly different. * *p* < 0.05, ** *p* < 0.01.

We further looked for bacterial OTUs that showed a significant association with specific *Phlebotomus* species and, hence, were responsible for the observed distinct clustering of the bacterial communities (Figure 4). OTUs 1, 2 and 159, belonging to *Spiroplasma, Wolbachia* and *Spiroplasma* respectively, showed significantly higher RA (*p* < 0.001) in *P. papatasi* (Figure 5), particularly the first two which were the most abundant OTUs in our study. OTUs 3, 263, 284, 391 and 720, belonging to *Wolbachia, Pseudomonadaceae, Xanthomonadaceae* (284 and 391) and *Brachybacterium,* respectively, showed significantly higher RA (*p* < 0.05) in *P. neglectus* (Figure 5). OTUs 18 and 295, assigned to γ-proteobacteria and *Rhodobacteriaceae,* respectively, showed significantly higher RA (*p* < 0.05) in *P. similis* compared to the other *Phlebotomus* species (Figure 6). Finally, OTUs 12, 154, 204 και 255, assigned to *Pseudomonas, Flavobacterium, Saprospiraceae* and *Acinetobacter*, respectively, showed significantly higher RA (*p* < 0.05) in *P. tobbi* (Figure 5). We further observed several OTUs that were either disfavored in a certain *Phlebotomus* species or they were associated with more than one species. In the former case, OTUs 5, 37 and 45, assigned to *Staphylococcus, Micrococcus* and *Corynebacteriaceae,* respectively, showed significantly lower RA (*p* < 0.05) in *P. papatasi* compared to the other three species (Figure 6). On the other hand, OTUs 7, 11 and 78, belonging to *Cutlbacterium, Rhizobiaceae* and *Pseudomonas,* respectively, showed significantly higher RA (*p* < 0.05) in *P. similis* and *P. neglectus*, while OTUs 220 and 471, belonging to the Actinobacterial genera *Brevibacterium* and *Corynebacterium,* respectively, were associated (*p* < 0.05) with *P. neglectus* and *P. similis* (Figure 5).

We also explored the presence of archaea in *Phlebotomus* species. Archaeal OTUs showed low overall RA compared to bacteria. The five most abundant OTUs belonged to Nanoarchaeota (*Woesearchaeia*), Thaumarchaeota (*Nitrososphaeraceae*), and Eyrarchaeota (*Methanobacterium*) with RA ranging from 0.002% to 0.006% (Figure 6). Correlation testing showed that OTU_503, assigned to *Nitrosphaeraceae*, being the second most abundant archaeal OTU, showed an exclusive presence in *P. similis*. Similarly, OTU_1309 assigned to *Methanobrevibacter*, showed significantly higher RA in *P. neglectus* compared to *P. papatasi* and *P. tobbi.*

## 3. Discussion

In this study we determined the composition of the gut prokaryotic microbiota of *Phlebotomus* species and explored the role of host phylogeny in structuring the gut bacterial and archaeal community. The sandfly population collected from the island of Leros was composed of 10 species, with *P. papatasi, P. neglectus* and *P. tobbi* being the most dominant, followed by *S. minuta* and *P. simils*, representing the sandfly fauna on the island of Leros, Greece. Previous studies in Serbia, Albania and other countries in the East Mediterranean basin have shown similar sandfly species distribution with *P. neglectus* most often identified as the dominant species [7,8,9,10]. A recent study in the same study region in Greece reported *P. neglectus* as the predominant species, followed by *S. minuta, P. tobbi, P. simici* and *P. similis* [12], which were also detected in our study. Additionally, in the current study we report the presence of *P. papatasi, P. perfiliewi, P. mascitti* and *P. alexandri* on the island. Discrepancies between monitoring surveys of sandfly populations performed in the same region at different time periods is a rather common observation and could be attributed to: (i) different sampling periods (i.e., in [12] samples were collected only during 3 sampling days in one sandfly season vs. several occasions in two consecutive sandfly seasons) and (ii) different sampling microhabitats (*P. neglectus* and *P. papatasi* have been reported to have different optima of relative humidity (RH) and temperature (RH 50%–60%, 27–29 ℃ vs. RH 30%–40%, 25–27 ℃) [12]. In line with this, Alten et al., [7] showed that the population density of *P. neglectus* in a given region could vary substantially from year to year as a function of varying environmental conditions.

The gut microbiota of the *Phlebotomus* species was dominated by Proteobacteria, mostly α- and γ-proteobacteria, Firmicutes, Tenericutes and Actinobacteria. This is in line with previous studies in wild caught sandflies [28,29,37] and other insects [25,38].

Most previous microbiota studies on *Phlebotomus* have used culture-dependent approaches [28,29,39,40], with their well-documented limitations [41], and did not explore the factors shaping the gut microbial community of wild populations of *Phlebotomus* sandflies. Previous studies have highlighted, among other factors, the role of host phylogeny on the composition of insect gut microbial community [24,42,43]. In this context, we explored the role of host species on the diversity of the gut microbiota of a wild population of *Phlebotomus* sandflies. In order to focus on host genotype effects, we only considered in our study *Leishmania*-free specimens. We noted a clear differentiation of the gut microbiota at the α-diversity level between *P. similis* and *P. tobbi*, dominated by several low-abundance members, and *P. neglectus* and *P. papatasi* whose bacteriome was dominated by a few highly prevalent OTUs. Multivariate analysis at the β-diversity level showed that all *Phlebotomus* species carried distinct microbial communities, with *P. papatasi* showing a more distinct bacterial community compared to the other three species, which also differed to each other to a lower extent. These findings verify our initial hypothesis that, in the absence of *Leishmania,* host phylogeny has a significant structural role in gut bacterial community assembly of *Phlebotomus* species. 

The distinct composition of the bacterial community of *P. papatasi* was driven by three OTUs belonging to *Spiroplasma* and *Wolbachia*. These are common facultative endosymbionts of *Phlebotomus* [28,44], *Lutzomyia* [30,31] and other insects; it is now estimated that *Wolbachia* and *Spiroplasma* endosymbionts are present in up to 30% of all insects [45]. They are maternally inherited, and they affect host ecology, physiology and fitness [15,46,47]. Their extensive co-presence in *P. papatasi* contrasts previous studies in sandflies [44] and ants [48] which have suggested that their co-detection in the same host is a rather infrequent event. Similarly, the microbiota of *P. neglectus* was overwhelmed by another *Wolbachia,* shared with *P. tobbi,* and a *Staphylococcus* shared with *P. tobbi* and *P. similis*. *Staphylococcus* have been reported as common dwellers of the gut of *L. longipalpis* [49,50], *L. evansi* [37] and *P. papatasi* [28], although here they were specifically absent from this species. *Staphylococcus* have been incriminated as pathogens of various organisms including insects [51], although their role in the biology of insect hosts remains unknown. Overall, the high abundance of *Wolbachia* and *Spiroplasma* in the female *Phlebotomus* fauna in the study area might have serious, positive or negative, implications for the sandfly population depending on the type of effect these endosymbionts impose on their host. Further investigations will focus on the role of these symbionts on the biology of the *Phlebotomus* species considering the contrasting evidence for the role of endosymbiotic bacteria on the infectivity and survival of *Leishmania* [27,52]. 

The gut of *P. tobbi* and *P. similis* were co-colonized by bacteria showing low relative abundance (1%–4%) and belonged to *Cutibacterium, Rhizobiaceae* and *Pseudomonas*. *Cutibacterium* are common members of the insect gut microbiota of *Bactocera oleae* at early developmental stages [53]. They are mostly known as early colonizers of infants where they function as lactate-consumer and propionate-producer [54]. Their presence in the gut of sandflies might be associated with feeding and digestion mechanisms. *Rhizobiaceae* encompass a range of bacterial genera which are prevalent in soil and plant tissues, hence their presence in insect gut has been associated with plant feeding lifestyles [55,56]. *Pseudomonas* are common dwellers of the gut of wild caught sandflies [4,30,57]. Their common presence in larvae and adults of sandflies was attributed to acquisition by soil during the soil feeding larvae stage [37]. However recent studies with laboratory-fed sandflies showed that *Pseudomonadaceae* constitute significant members of the gut microbiota of *L. longipalpis* with their abundance depending on the meal, plant sugar or blood [26,27]. 

With the applied methods used we were also able to determine the archaeal community in sandflies gut. The contribution of archaea in the prokaryotic microbiota of sandflies was low (<0.01%), compared to termites (3%), whose archaeal symbiotic community has been thoroughly studied [58]. This underrepresentation of archaea in most studies, including ours, could be attributed either to the use of universal bacterial primers which was recently shown to be biased towards bacteria in the human gut microbiota [59], the lack of well populated 16S rRNA reference databases or indeed the low abundance of archaea in the studied samples [60]. Considering the lack of previous studies on sandflies, we provide first evidence for the presence of archaea belonging to Nanoarchaeota, methanogenic Euryarchaeota and Thaumarchaeota in the gut of *Phlebotomus*. Methanogenic Euryarchaeota belonging to *Methanobrevibacter, Methanobactarium* and *Methanosarcina*, all being obligate anaerobes, are common dwellers of the gut of termites [32,58] and Coleoptera [61], producing methane from CO_2_/H_2_ or formate. Their presence might suggest the establishment of anaerobic zones in the gut of *Phlebotomus*. Thaumarchaeota of the family *Nitrososphaeraceae* have been also detected in the gut of beetles [61]. They are ubiquitous in soil, plants and water ecosystems responsible for the oxidation of ammonium to nitrite [62]. However, their exact role in the gut of sandflies warrants further investigation. However, the most abundant archaeal OTU belonged to Nanoarchaeota, previously detected in the human lung [59], but reported for the first time in an insect gut. Nanoarchaeota are obligate symbionts of Crenarchaeota [63,64]. They are characterized by a reduced genome, hence relying on their host for central cellular biosyntheses [65]. Their detection in diverse aquatic and terrestrial ecosystems [66,67,68,69] suggests a rapidly expanding range of hosts and their dispersal in new environments. Nanoarchaeota could exert deleterious effects on their host like *N. equitans,* which prevents host replication [70], while terrestrial members do not seem to be deleterious to their host and might be beneficial under certain conditions [64].

## 4. Materials and Methods 

### 4.1. Samples Collection

Sandflies were collected from the island of Leros in the south-eastern area of the Aegean Sea, Greece (Latitude: 37°08′60.00″ N, Longitude: 26°50′59.99″ E), a previously reported habitat of several *Phlebotomus* species [12]. The total area of the island is 54 km² and the natural environment is typical Mediterranean (Supplementary Materials Figure S4). 

Sand fly collection was performed by using Centre for Disease Control (CDC) miniature light traps and BG Sentinel traps at regular intervals for two sandfly activity seasons (April to October of 2017 and 2018). Traps were placed in domestic and peridomestic environments nearby animal sheds with cattle, goats, sheep, or poultry to maximize trapping capacity [71]. The traps were operated all night and the sandflies were collected early in the morning, sorted, and placed in Eppendorf tubes in 80% ethanol before being transported to the laboratory for downstream processing and analyses. 

### 4.2. Species-Level Identification of Phlebotomus Sandflies 

The head and the rear part of the abdomen of all collected sandflies were mounted on permanent microscope slides and species identification was carried out based on the morphology of the pharynx, male genitalia or female spermathecae [72,73]. The stomach and the gut of each dissected specimen were then stored together at −80 °C for DNA extraction. 

### 4.3. DNA Extraction 

Upon species identification female sandflies of the same species, collected at the same time point from the same trap were homogenized in groups of 5. DNA was extracted from homogenized samples using the DNeasy Blood and Tissue Kit (Qiagen GmbH, Hilden, Germany) following the manufacturers’ protocol. The size and the integrity of the DNA extracted was verified by agarose gel electrophoresis (1%) and DNA was quantified using a Qubit fluorometer with a Quant-iT HS double-stranded DNA (dsDNA) assay kit (Invitrogen, Carlsbad, CA, USA). 

### 4.4. Quantitative Polymerase Chain Reaction (q-PCR) Detection of Leishmania spp. in Sandfly Samples

All samples were tested for the presence of *L. infantum* with a TaqMan real time qPCR assay, targeting a 120 bp fragment of the kinetoplast minicircle DNA as reported before [74].

### 4.5. PCR Amplification of the 16S rRNA Gene and Amplicon Sequencing Analysis of the Microbiome

The composition of the bacterial and archaeal community (co-amplified with the primer sets and protocol used) was determined with amplicon sequencing of the 16S rRNA gene via HiSeq Illumina Rapid Mode 2 × 250 bp paired-end reads (Illumina Inc., San Diego, CA, USA) in the DNA Sequencing Center, Department of Biology, Brigham Young University (GSC-BYU, Provo, UT, US). Bacterial and archaeal 16S rRNA genes were amplified with the primer set 515f-806r [75,76], targeting the V4 region of the 16S rRNA gene, following the protocol of the Earth Microbiome Project [77]. For all PCR amplifications, the Q5® High-Fidelity DNA Polymerase (NEB, Massachusetts, USA) was used. All samples were initially amplified (28 amplification cycles) using the domain-specific primers mentioned above, followed by a PCR (7 amplification cycles) using the same primers but this time the forward primer 515r (5’-TTXXXXXXXXXGTGTGYCAGCMGCCGCGGTAA-3’) carried 5’ extensions comprising of linkers (italics) and indexes (underlined) used for samples barcoding for multiplex sequencing. PCR conditions are listed in Supplementary Materials Table S1.

### 4.6. Bioinformatic Analysis of Amplicon Sequencing Data 

The amplicon sequencing data were analyzed as described by Katsoula et al., [78]. Briefly, the raw sequence data were demultiplexed with Flexbar v3.0 [79]. The reads were quality trimmed with Trimmomatic v0.32 [80] for a length cutoff of 150 bp and were assembled with FLASH v1.2.8 [81] using the default parameters unless otherwise stated. OTU calling at 97% identities was performed with the UPARSE v10.0.240 software [82]. Chimeric sequences were identified with the UCHIME v4.2 software [83] using the RDP Gold database vMicrobiomeutil-r20110519. Sequence classification was performed with Lambda v0.9.1 [84] against the Silva v128 small ribosomal subunit database [85]. Misclassified sequences were removed from downstream analysis.

### 4.7. Statistical Analysis 

The OTU matrix of bacteria was used to assess the effect of host genotype on the α- and β-diversity. The impact on the α-diversity was determined via calculation of the diversity indices richness (S), Fisher alpha, inverse Simpson, Shannon [86] and Pielou’s evenness [87] and the data were subjected to one-way analysis of variance (ANOVA). Differences in the β-diversity of the bacterial community between different *Phlebotomus* species were modelled with CCA. We further used pairwise permutational multivariate analysis of variance (PERMANOVA) to identify significant differences in the structure of the bacterial communities between different sandfly species [88]. All statistical analyses were performed with the R v3.5.2 software [89]. The data were submitted to the Sequence Read Archive of the National Center for Biotechnological Information (NCBI) with bioproject accession number PRJNA630369.

## 5. Conclusions

The gut prokaryotic microbiota of a wild, *Leishmania*-free, population of *Phlebotomus* sandflies composed of four main species was structured according to host genotype. Endosymbiotic *Wolbachia* and *Spiroplasma* seemed to dominate the bacterial community of *P. papatasi* and *P. neglectus*, while *P. tobbi* and *P. similis* supported a more diverse bacterial community composed of several less-abundant bacteria. Archaea were minor members of the prokaryotic microbiota of sandflies with methanogenic Euryarchaeota, nitrifying Thaumarchaeota and obligate symbiotic Nanoarchaeota being most prevalent. Our study provides the first high-resolution analysis of the bacterial gut microbiota in *Phlebotomus*, which is structured (in the absence of *Leishmania*) according to host phylogeny, and reports pioneering evidence for the presence of archaea in the gut of sandflies. Further studies will explore the role of *Leishmania* in combination with biogeography and host genotype on the assembly of the gut microbial community of wild populations of *Phlebotomus* sandfies.

## Supplementary Materials

The following are available online at https://www.mdpi.com/2076-0817/9/6/428/s1: Figure S1: The standard curve (a), amplification curve (b) and dissociation curves (c) of the quantitative polymerase chain reaction (q-PCR) measurements employed for the detection and quantification of *Leishmania infantum* in sandfly DNA samples. Only standard solutions used for the preparation of the standard curve were positive in amplification as indicated in the amplification and dissociation curves, Figure S2: Rarefaction curves indicating the depth of diversity coverage of the bacterial community in the sandfly samples analysed, Figure S3: Heatmap representing the OTUs composing the core microbiota of *Phlebotomus* species analysed in the current study. OTUs were characterized as core microbiota members if they participated with at least 0.1% in at least the 50% of the samples derived from the insect of interest, Figure S4: The geographic location of the study area where sandfly samples were collected, Table S1: PCR reagents and thermocycling conditions used for amplicon sequencing analysis.
