# Supplementary material for: Host Species Determines the Composition of the Prokaryotic Microbiota in Phlebotomus Sandflies
Source: Pathogens. 2020 May 29;9(6):428. doi: 10.3390/pathogens9060428 (PMC7350354; doi:10.3390/pathogens9060428)
Supplement: Supplementary file 1 [file pathogens-09-00428-s001.zip › Supplementary Figures Papadopoulos et al., 8.5.2020.pptx]

## Slide 1
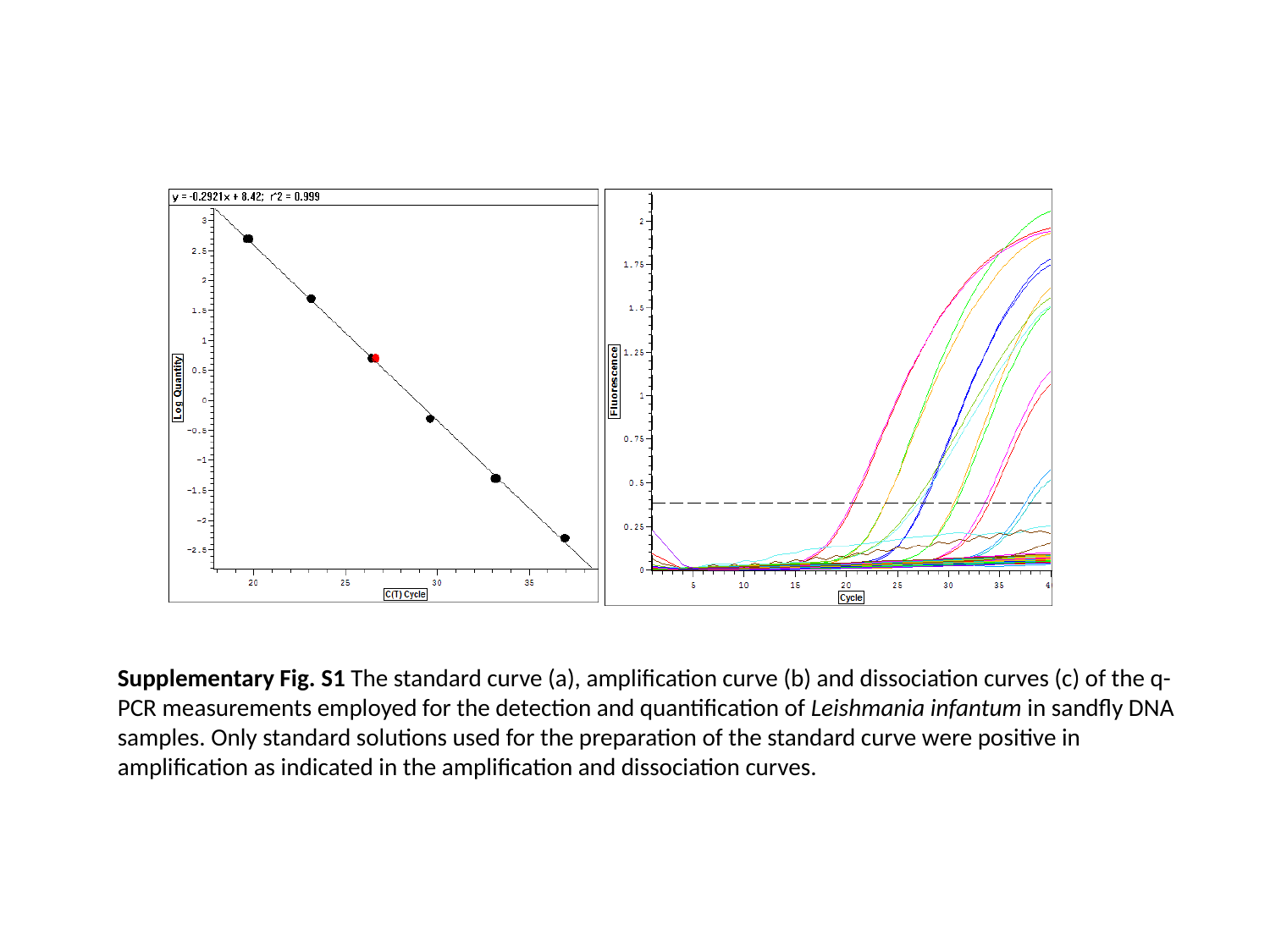

Supplementary Fig. S1 The standard curve (a), amplification curve (b) and dissociation curves (c) of the q-PCR measurements employed for the detection and quantification of Leishmania infantum in sandfly DNA samples. Only standard solutions used for the preparation of the standard curve were positive in amplification as indicated in the amplification and dissociation curves.

## Slide 2
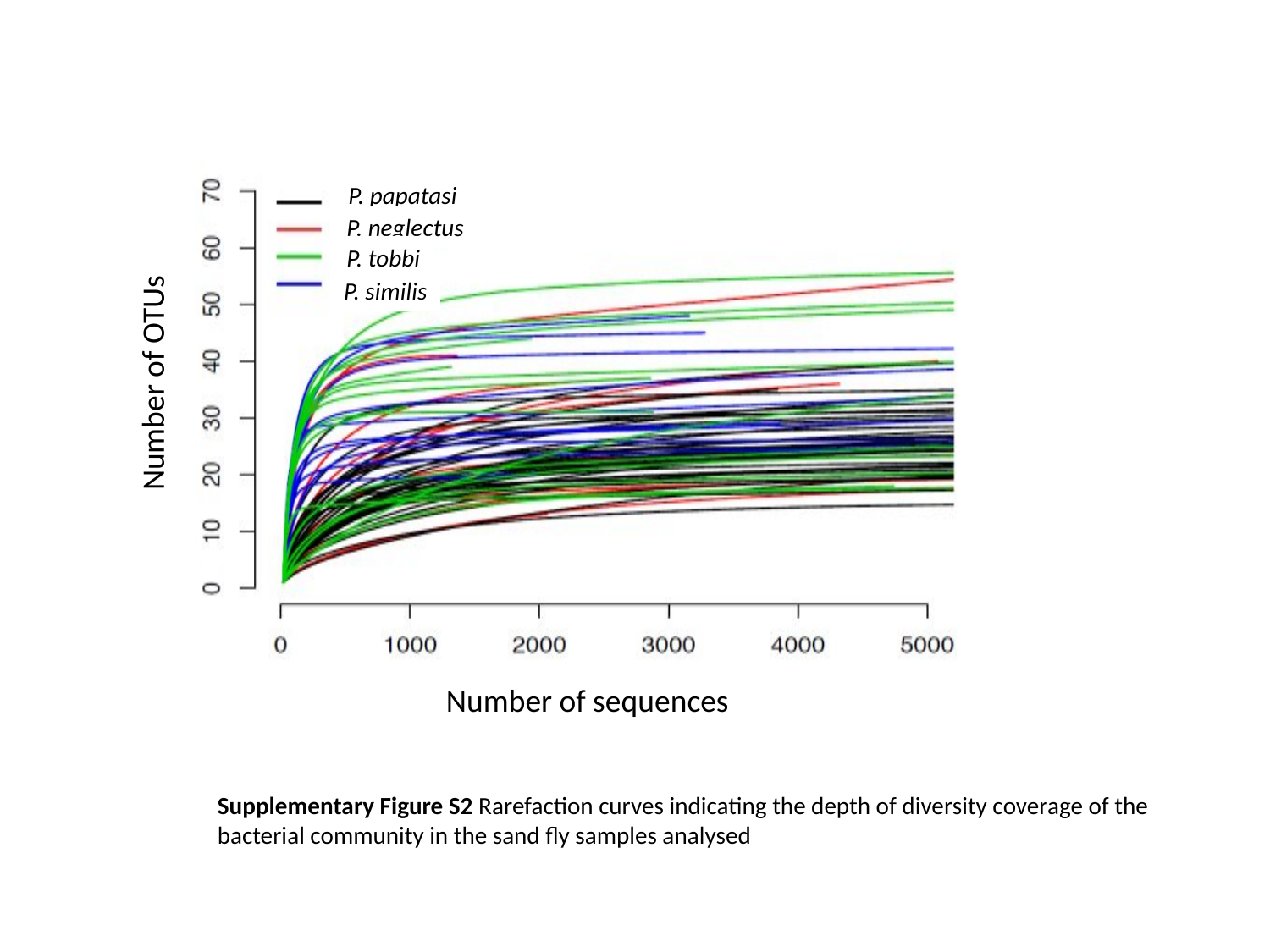

P. papatasi
P. neglectus
P. tobbi
P. similis
Number of OTUs
Number of sequences
Supplementary Figure S2 Rarefaction curves indicating the depth of diversity coverage of the bacterial community in the sand fly samples analysed

## Slide 3
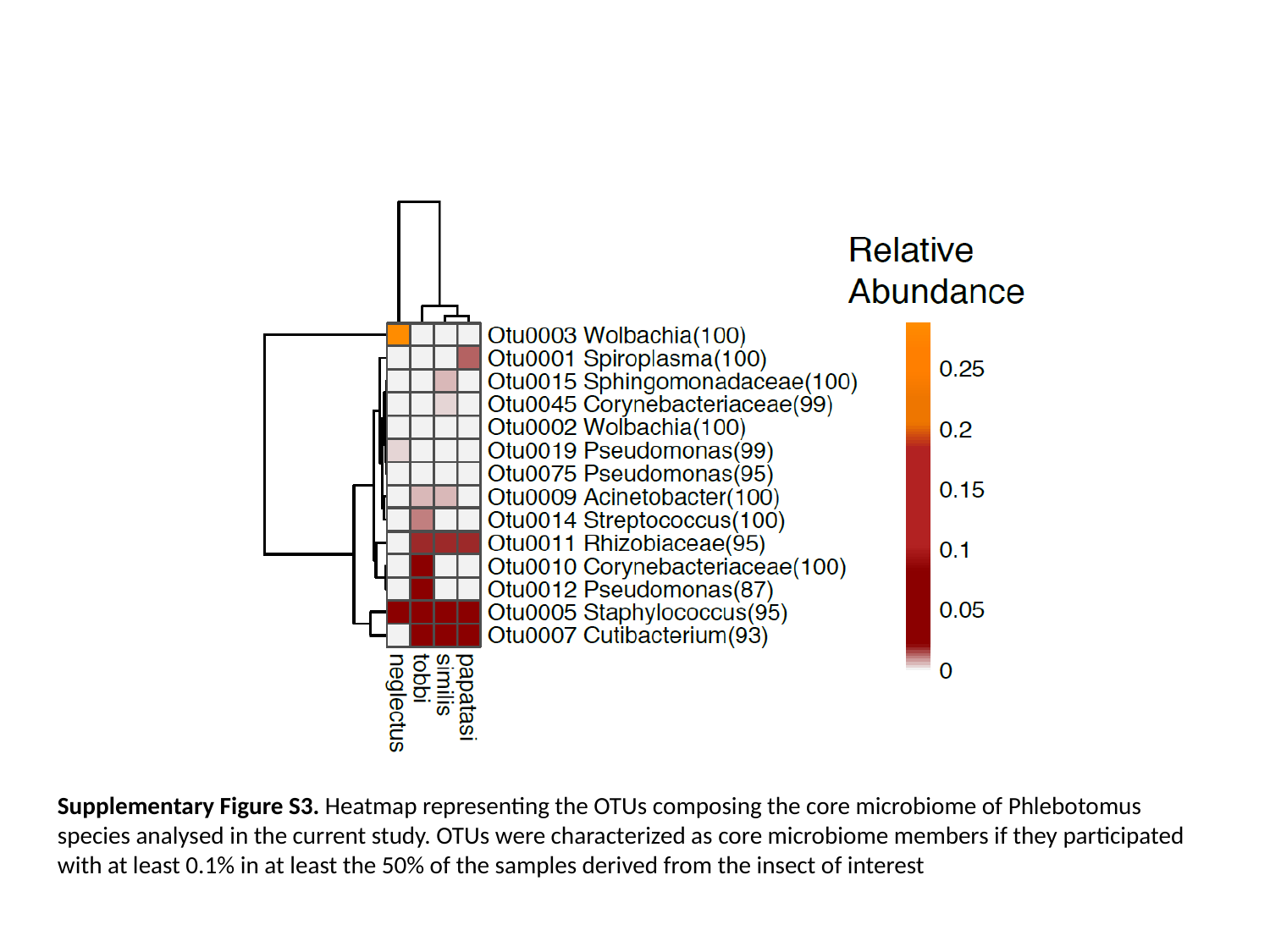

Supplementary Figure S3. Heatmap representing the OTUs composing the core microbiome of Phlebotomus species analysed in the current study. OTUs were characterized as core microbiome members if they participated with at least 0.1% in at least the 50% of the samples derived from the insect of interest

## Slide 4
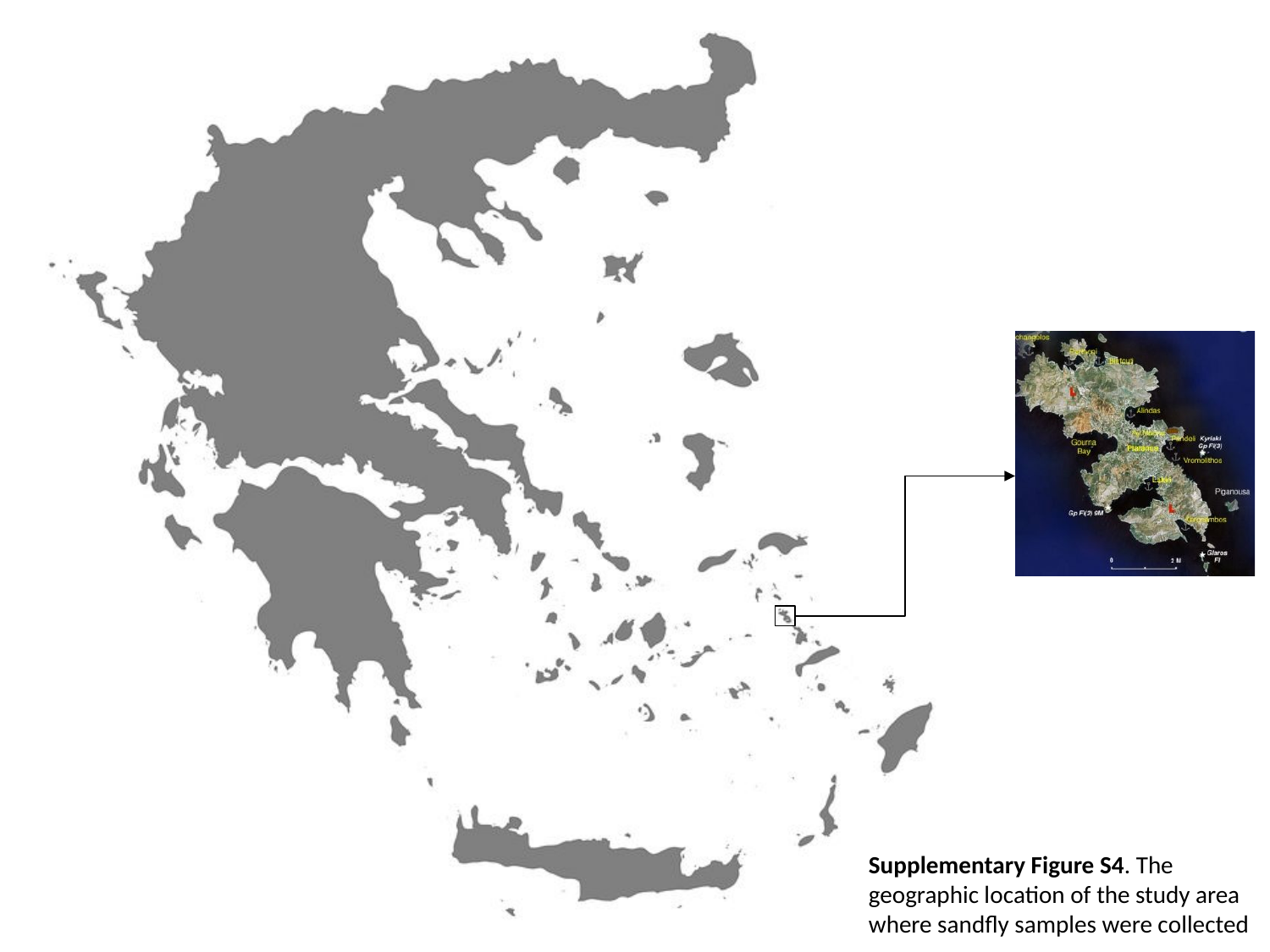

Supplementary Figure S4. The geographic location of the study area where sandfly samples were collected
