# Supplementary material for: Host Species Determines the Composition of the Prokaryotic Microbiota in Phlebotomus Sandflies
Source: Pathogens. 2020 May 29;9(6):428. doi: 10.3390/pathogens9060428 (PMC7350354; doi:10.3390/pathogens9060428)
Supplement: Supplementary file 1 [file pathogens-09-00428-s001.zip › Supplementary Table S1 Papadopoulos et al., 2020.docx]

**Supplementary Table S1.** PCR reagents and thermocycling conditions used for amplicon sequencing analysis.

| **PCR reaction** | | |  |
| --- | --- | --- | --- |
| **Reagents** | **Volume (μl)** | **Concentration** | **Comments** |
| Primer F | 1 | 0.5 μΜ |  |
| Primer R | 1 | 0.5 μΜ |  |
| BSA | 0.4 | 0.4 μg/μl | Added only in the first amplification step |
| Polymerase Q5 (2x MasterMix) | 10 | 1x |  |
| ddH_2_O | 5.6 |  |  |
| DNA | 2 | 0.2 ng/μl |  |
| Total | 20 |  |  |
| **PCR conditions** | | | |
| **Step** | **Temperature (°C)** | **Time** | **Number of Cycles** |
| Initial Denaturation | 98 | 30 sec |  |
| Denaturation | 98 | 10 sec | 28 in the first amplification step / 7 in the second amplification step |
| Annealing | 50 for bacteria | 30 sec |  |
| Extension | 72 | 30 sec |  |
| Final extension | 72 | 10 min |  |
